# Surface Area of Patellar Facets: Inferential Statistics in the Iraqi Population

**DOI:** 10.1155/2017/2685159

**Published:** 2017-02-28

**Authors:** Ahmed Al-Imam, Zaid Al-Zamili, Rawan Omar

**Affiliations:** ^1^Department of Anatomy and Cellular Biology, Faculty of Medicine, University of Baghdad, Baghdad, Iraq; ^2^Novel Psychoactive Substances Research Unit, University of Hertfordshire, Hatfield, UK; ^3^Faculty of Medicine, University of Baghdad, Baghdad, Iraq

## Abstract

*Background*. The patella is the largest sesamoid bone in the body; its three-dimensional complexity necessitates biomechanical perfection. Numerous pathologies occur at the patellofemoral unit which may end in degenerative changes. This study aims to test the presence of statistical correlation between the surface areas of patellar facets and other patellar morphometric parameters.* Materials and Methods*. Forty dry human patellae were studied. The morphometry of each patella was measured using a digital Vernier Caliper, electronic balance, and image analyses software known as ImageJ. The patellar facetal surface area was correlated with patellar weight, height, width, and thickness*. Results*. Inferential statistics proved the existence of linear correlation of total facetal surface area and patellar weight, height, width, and thickness. The correlation was strongest for surface area versus patellar weight. The lateral facetal area was found persistently larger than the medial facetal area, the *p value* was found to be <0.001 (one-tailed* t*-test) for right patellae, and another significant *p value* of < 0.001 (one-tailed* t*-test) was found for left patellae.* Conclusion*. These data are vital for the restoration of the normal biomechanics of the patellofemoral unit; these are to be consulted during knee surgeries and implant designs and can be of an indispensable anthropometric, interethnic, and biometric value.

## 1. Introduction

**Figure 1:**
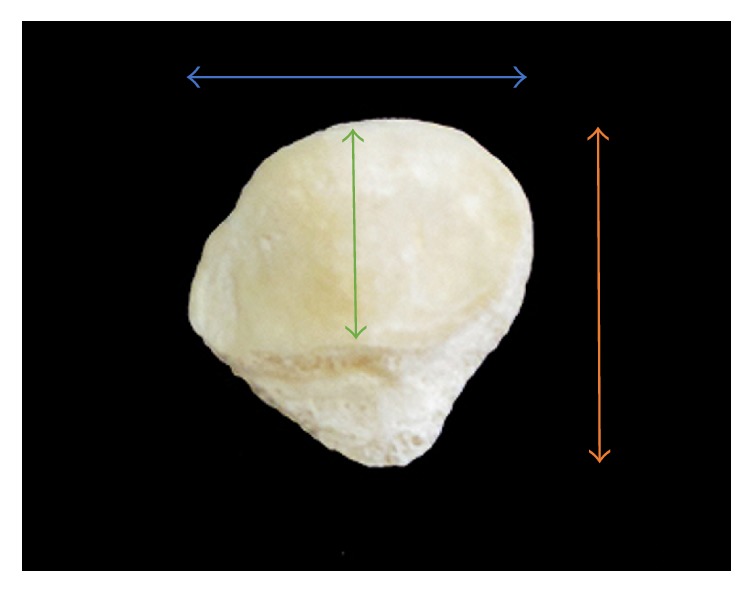
Posterior view of the patella. *H*1 (vertical orange line), *H*2 (vertical green line), and *W* (horizontal blue line).

The patella is the largest sesamoid bone in the body which develops within the tendon of the quadriceps femoris muscle. The patella is a flat bone of a shield-like morphology (Figure 1); it is located anterior to the femoral condyles. It has two surfaces (anterior and posterior), three borders (superior, medial, and lateral), and an apex. The posterior surface of the patella is divided into two parts: superior (articular) and inferior (nonarticular). The articular surface is further subdivided into medial and lateral facets, separated by a vertical ridge [1]. The patella is a bone of an anthropometric and biometric significance. It is elaborately involved in a variety of sitting and squatting postures. Hence, its irregular-complex morphology and dimensions are liable for modification by environmental factors, including ethnic and cultural variables [2].

As a rule, it has been assumed that the size of the patella can be dependent on the strain generated by the quadriceps muscle. However, the absence of patella in some animals which have a very powerful capacity of knee extension led to the controversy concerning this assumption [2]. Yoo et al. (2007) revealed that the geometry of the patella and the patellar tendon was larger in males. Other demographic factors including weight, height, and body mass index, all, correlate well with patellar thickness [3, 4]. Potage et al. (2015) demonstrated that the quadriceps tendon thickness is positively correlated with the patellar height [5]. Koyuncu et al. (2011) studied the patellar development during the fetal life. It was concluded that there are no significant differences between genders or sides (right versus left patellae). However, a significant correlation was found between gestational age and all studied morphometric parameters of patella [6].

Examination of a southern Chinese population revealed that males have larger patellae, and there was no statistically significant difference between right versus left patellae. Compared with Westerners, these patellae were smaller [7]. In northern Indian population, a study used patellar morphometry for determination of sex; the results were of high accuracy up to 80.5% [8]. Olateju et al. (2013) studied a South African population of European ancestry, revealing a high positive linear correlation (*R *= 0.89) between the patellar thickness and width [1].

In this study,* patellar facet angle* (PFA) was not measured. However, it is worth mentioning that other studies about patellofemoral unit surgeries indicated that there was a relationship between the preoperative PFA and the subsequent postoperative osteosclerosis of the unit. Therefore, both patellar morphometry and patellofemoral implant design(s) can influence the physical stress distribution within the unit and particularly following* total knee arthroplasty* (TKA) [9–14].

Numerous pathologies can affect the patellofemoral compartment of the knee joint like osteoarthritis, chondromalacia patellae, fractures and stress fractures, idiopathic patellofemoral pain syndrome, and others [15]. Patients who are undergoing TKA are routinely subjected to a predetermined amount of postsurgical loading of the knee, to preserve patellar articular cartilage and subchondral bone integrity [16]. Other pathologies that may affect the patella during TKA include patellofemoral instability and implant's failure [17]. Biomechanical disturbances can also affect the patellofemoral compartment of the knee, for example, when the Q-angle is larger than normal; there will be overpressure on the medial knee compartment during manoeuvres that increase contact between the patella and medial condylar facet [18, 19].

This study was authorised by the ethical approval numbered 620-73, dated on 15 May 2016. The approval was authorised by the ethical approval committee of the Faculty of Medicine at the University of Baghdad. The level of evidence of this paper is level 5; the categorization is based upon the classification system by the Oxford Centre for Evidence-Based Medicine [20].

## 2. Materials and Methods

Materials used included 40 human patellae of the Iraqi ethnicity; both age and gender were unknown. The patellae included 20 right patellae and 20 left patellae. These patellae belonged to deceased members of the Iraqi ethnicity. The measured morphometric parameters for each patella included two patellar heights measurement (*H*1, *H*2), the maximal width (*W*) measured perpendicularly to the midpoint of the main facetal vertical ridge (Figure 1), and the maximal thickness (*T*). The maximal thickness was measured at the level of the midpoint of the facetal ridge. The surface area of each of the two (lateral and medial) main patellar facets was measured using digital imaging analysis software known as ImageJ software [21]; these area measurements were accurate down to a thousandth of a square centimetre (cm^2^). Additionally, the weight of each patella was measured using an electronic balance; these measurements were also accurate to the nearest thousandth of a gramme (gm).

Measurements were taken at the Anatomical Specimens' Unit at the Department of Anatomy and Cellular Biology. Further, to prevent human-made errors and biases while measuring, two of the authors of this paper recorded each morphometric parameter twice. Linear measurements (*H*1, *H*2, *W*, and *T*) were done using a standard digital electronic Vernier Caliper (Vernier micrometre gauge tool, UPC number 814870023454), and the unit of tabulated data (Table 1) is the millimetre (mm). When the two measurements were different to the nearest 1/10th of a millimetre, a 3rd independent reading was taken to resolve the numerical disparity. This step was followed by taking the average numerical value for each of the measured morphometric parameters (Table 1). Statistical analyses, descriptive (Table 2) and inferential, were done by using the* Statistical Package for Social Sciences* (SPSS 22) and Microsoft Excel 2016. Inferential statistics included the application of analysis of variance (ANOVA), linear regression models, and Student's* t*-test for paired and unpaired data. Statistical inference, at an alpha value (*α*) of 0.05, was considered to test the level of significance.

A systematic review of the literature was done from 2 August 2016 to the 18 November 2016, to collect the most valid and up-to-date literature on the topic. The literature review was done across medical databases: PubMed, the Cochrane Library, Scopus, Google Scholar, and unpublished literature (grey literature). Prespecified keywords were used, in combination with Boolean operators [22]. The total number of papers pertinent to the study topic, after exclusion of duplicate articles, was 37 papers. These papers were scanned and filtered, after a detailed inspection of each paper's title, abstract, and the main body of the manuscript. The papers were also assessed for their level of evidence using critical appraisal tools [23].

## 3. Results and Discussion

**Figure 2:**
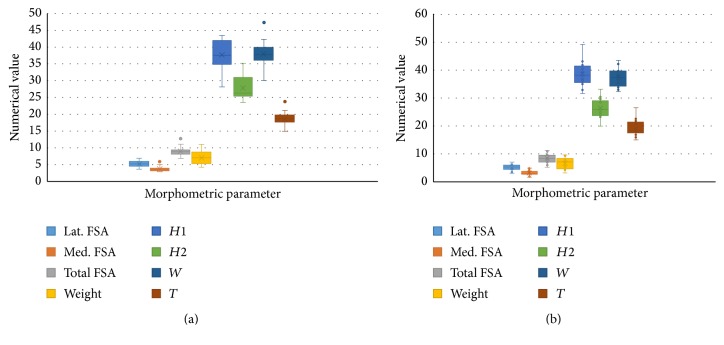
Box-and-Whisker Plot: right patellae (above) versus left patellae (below). ^#^FSA = facetal surface area.

Descriptive statistics including measures of central tendency and plotting a Box-and-Whisker Plot (Figure 2) revealed that the data follow a normal distribution pattern. Hence, parametric statistical tests applicable for continuous numerical data were employed. Based on descriptive statistical data (Table 1) and in relation to the patellar facetal surface area, the mean value of the lateral facetal surface area of right patellae was 5.25 cm^2^ (±) 0.91, while the mean value of the lateral facetal surface area of the left patellae was 5.35 cm^2^ (±) 1.16. On the other hand, the mean value of the medial facetal surface area of the right patellae was 3.74 cm^2^ (±) 0.71, while the mean value of the medial facetal surface area of the left patellae was 3.33 cm^2^ (±) 0.83. Apparently, the surface area of the lateral facet is larger than the surface area of the medial facet; this conclusion was consolidated by the application of inferential statistics: one-way analysis of variance (ANOVA) and Student's* t*-test. Both tests confirmed that the lateral facetal area was significantly larger than the medial facetal area, at a *p value* < 0.001 (one-tailed* t*-test) for right patellae and another significant *p value* of < 0.001 (one-tailed* t*-test) for left patellae. Furthermore, ANOVA test for the surface area of patellar facets (both medial and lateral) yielded a *p value* of < 0.001. All these correlations are highly statistically significant. Therefore, these data concord to the fact that the surface area of the lateral facet is larger than the medial facet; this seems to be the case in right patellae and left patellae.

On the other hand, there was no statistical difference between the surface areas of the same facet (medial or lateral) on different limbs. In other words, the surface area of lateral facet for both right and left patellae was not significantly different; the *p value* was 0.760 (2-tailed* t*-test). Similarly, the surface area of the medial facets of right and left patellae was not significantly different; the *p value* was 0.102 (2-tailed* t*-test).

**Figure 3:**
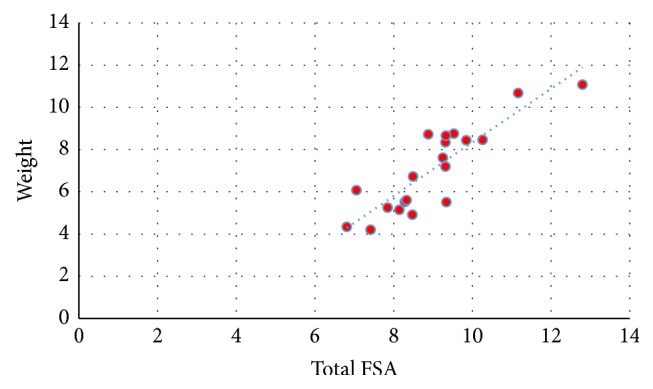
Scattered chart and linear regression; right patellae; total FSA versus weight. ^#^FSA = facetal surface area.

**Figure 4:**
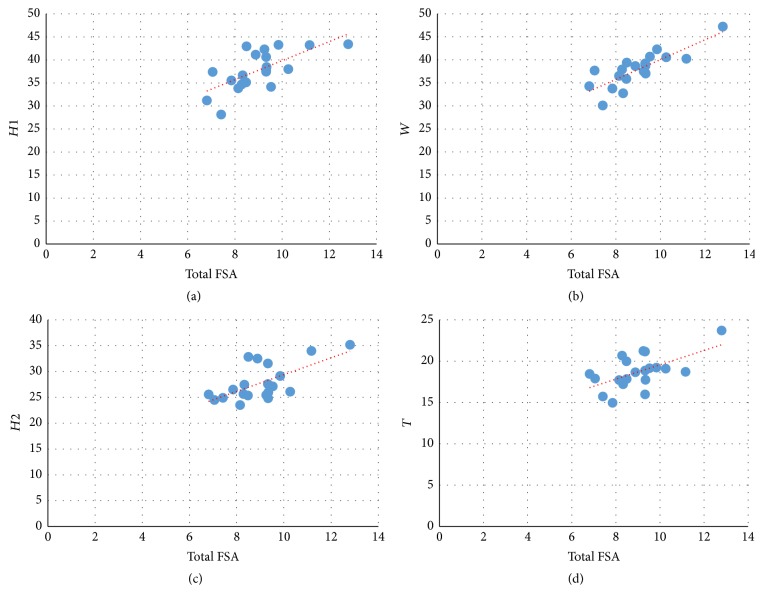
Scattered chart and linear regression, right patellae. Total FSA versus *H*1 (a), *H*2 (c), *W* (b), and *T* (d). ^#^FSA = facetal surface area.

**Figure 5:**
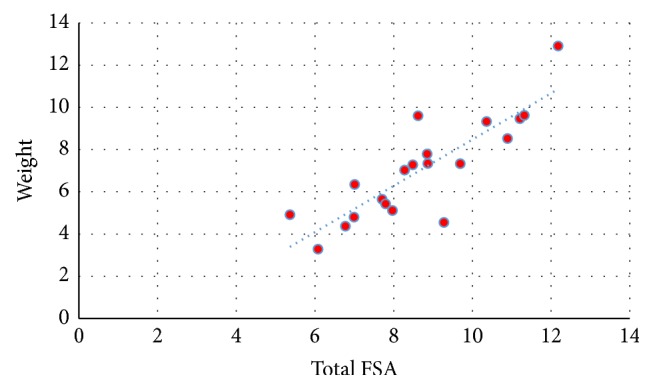
Scattered chart and linear regression, left patellae, total FSA versus weight. ^#^FSA = facetal surface area.

**Figure 6:**
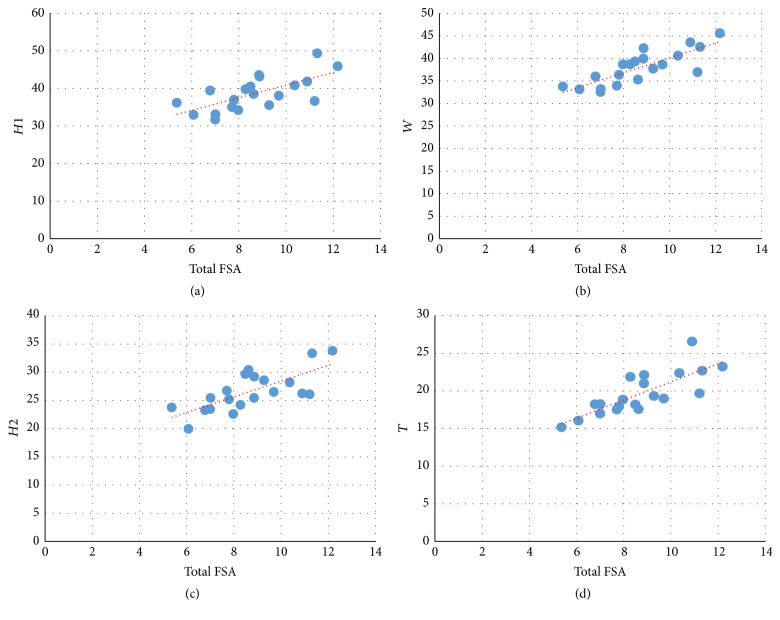
Scattered chart and linear regression, left patellae. Total FSA versus *H*1 (upper left), *H*2 (lower left), *W* (upper right), and *T* (lower right). ^#^FSA = facetal surface area.

Inferential statistical data using linear regression proved the existence of a positive linear correlation between the total patellar facetal surface area (total FSA) and patellar weight, height, width, and thickness (Figures 3–6). This pattern of linear correlation was applicable for both right and left patellae and with a comparable (similar)* r*-score and trend line inclination for patellae on both limbs. Interestingly, this pattern of linear correlation was the strongest for total FSA versus patellar weight, at a slope value of 1.269 for right patellae (Figure 3) and a slope value of 1.096 for left patellae (Figure 5). This pattern of correlation was more evident than with other morphometric parameters including height, width, and thickness for each patella (Figures 4 and 6).

It is worth mentioning that this study was carried out in a sample of patellae from individuals with unknown cerebral dominance patterns. Furthermore, the handedness (right-handed versus left-handed) of those individuals is unknown. Therefore, it is unknown if there are different patterns of correlation within the right-handed population in contrast to those who are left-handed. Al-Hadithi et al. (2016) studied the presence of a different pattern of visual analytics skills among individuals with different patterns of cerebral dominance and handedness [24, 25].

In summary, the lateral facetal surface was found to be larger than the medial facetal surface area and in both right and left patellae. On the other hand, there was no significant statistical difference between corresponding (similar) facets bilaterally. Total FSA was positively and linearly correlated with all other morphometric parameters including weight, height, width, and thickness. However, the correlation of total FSA with patellar weight was the most evident.

## 4. Conclusion

Several factors govern the morphology of the human patella; these are both genetic and environmental bases. Several pathologies that affect the patellofemoral unit may result in biomechanical dysfunction(s) of the unit itself or the knee joint as a whole. The restoration of normal patellofemoral morphology, morphometry, and biomechanics is mandatory to prevent superadded complications, including osteoarthritis of the knee and subsequent restriction of movement. This study, in the Iraqi population, provides original data in relation to the surface area of the patellar facets and the correlation between the surface area and other morphometric parameters of the patella, including the patellar weight, height, width, and thickness. Accordingly, it will be essential to restore the morphometry back to its original dimensions of the corresponding age, gender, and ethnicity. Furthermore, these data are applicable to implant design of the patella and patellofemoral unit. The aim is to conserve the functionality and persistence of the patellofemoral unit and the knee joint, which will maintain the mobility of the individual to the longest possible duration, and thereby enhance the quality of life and the lifespan of an individual.

## Figures and Tables

**(a):** 

Specimen^Ω^	Limb	Lat. FSA^#^	Med. FSA	Total FSA	Weight	*H*1	*H*2	*W*	*T*
(1)	Rt.	6.75	3.52	10.26	8.46	37.98	26.07	40.57	19.08
(2)	Rt.	5.96	3.57	9.53	8.75	34.15	27.10	40.71	19.14
(3)	Rt.	3.77	3.29	7.06	6.08	37.39	24.47	37.69	17.88
(4)	Rt.	6.90	5.90	12.80	11.07	43.42	35.16	47.24	23.71
(5)	Rt.	6.36	2.96	9.32	8.35	40.61	31.56	39.25	21.16
(6)	Rt.	5.51	4.33	9.84	8.44	43.25	29.11	42.31	19.22
(7)	Rt.	5.35	2.93	8.28	5.53	34.68	25.63	37.94	20.66
(8)	Rt.	3.67	3.14	6.81	4.34	31.17	25.54	34.31	18.44
(9)	Rt.	5.18	4.16	9.34	5.51	38.45	25.95	37.02	17.74
(10)	Rt.	5.34	3.98	9.32	7.20	37.71	27.57	37.21	15.98
(11)	Rt.	4.54	3.95	8.49	6.72	42.96	32.82	39.41	17.84
(12)	Rt.	4.31	3.53	7.85	5.26	35.54	26.49	33.78	14.95
(13)	Rt.	4.63	3.52	8.14	5.15	33.81	23.51	36.54	17.72
(14)	Rt.	4.20	3.22	7.41	4.21	28.11	24.91	30.11	15.71
(15)	Rt.	4.95	3.53	8.48	4.92	35.11	25.34	35.86	19.98
(16)	Rt.	5.79	3.46	9.25	7.61	42.31	25.41	37.51	21.22
(17)	Rt.	5.41	3.92	9.33	8.66	37.42	24.83	38.72	18.85
(18)	Rt.	5.15	3.18	8.33	5.60	36.64	27.43	32.77	17.19
(19)	Rt.	6.15	5.01	11.16	10.68	43.23	33.96	40.25	18.71
(20)	Rt.	5.11	3.77	8.88	8.72	41.15	32.48	38.68	18.65

^#^FSA = facetal surface area.

^Ω^Units of measurement are cm^2^ (surface area), gm (weight), and cm (length, width, and height).

**(b):** 

Specimen	Limb	Lat. FSA	Med. FSA	Total FSA	Weight	*H*1	*H*2	*W*	*T*
(1)	Lt.	7.77	4.41	12.18	12.91	45.92	33.79	45.56	23.22
(2)	Lt.	4.82	3.15	7.97	5.12	34.22	22.58	38.65	18.84
(3)	Lt.	6.59	3.77	10.36	9.33	40.81	28.20	40.62	22.36
(4)	Lt.	4.14	2.86	7.01	6.35	33.07	25.46	33.15	18.25
(5)	Lt.	5.56	3.72	9.28	4.55	35.57	28.57	37.71	19.31
(6)	Lt.	5.46	3.02	8.49	7.28	40.51	29.65	39.31	18.19
(7)	Lt.	5.18	3.10	8.28	7.03	39.81	24.21	38.65	21.85
(8)	Lt.	5.86	3.01	8.87	7.34	43.15	29.22	42.26	22.12
(9)	Lt.	6.36	3.34	9.69	7.33	38.07	26.49	38.61	18.98
(10)	Lt.	5.53	3.09	8.62	9.60	38.53	30.41	35.29	17.58
(11)	Lt.	6.22	4.98	11.21	9.47	36.65	26.07	36.95	19.65
(12)	Lt.	6.40	4.92	11.32	9.63	49.33	33.33	42.55	22.68
(13)	Lt.	4.57	2.42	6.99	4.80	31.74	23.44	32.56	16.98
(14)	Lt.	3.71	3.07	6.78	4.38	39.46	23.25	35.98	18.21
(15)	Lt.	4.24	1.83	6.08	3.28	32.97	19.94	33.13	16.05
(16)	Lt.	4.52	3.19	7.71	5.65	35.04	26.72	33.96	17.52
(17)	Lt.	4.73	4.12	8.85	7.79	43.54	25.43	39.93	20.99
(18)	Lt.	3.23	2.13	5.36	4.91	36.18	23.74	33.77	15.17
(19)	Lt.	7.14	3.75	10.90	8.53	41.82	26.24	43.54	26.55
(20)	Lt.	5.01	2.79	7.79	5.43	36.97	25.17	36.35	17.91

**(a):** 

Ω	Lateral FSA^#^	Medial FSA	Total FSA	Weight	*H*1	*H*2	TD	*T*
Mean	5.25	3.74	8.99	7.06	37.75	27.57	37.89	18.69
Median	5.26	3.53	9.06	6.96	37.57	26.07	37.82	18.68
Variance	0.82	0.51	1.94	4.12	18.32	12.05	13.52	4.19
St. Dev.	0.91	0.71	1.39	2.03	4.28	3.47	3.68	2.05

^#^FSA = facetal surface area.

^Ω^Units of measurement are cm^2^ (surface area), gm (weight), and cm (length, width, and height).

**(b):** 

	Lateral FSA	Medial FSA	Total FSA	Weight	*H*1	*H*2	TD	*T*
Mean	5.35	3.33	8.69	7.04	38.67	26.43	37.93	19.69
Median	5.32	3.13	8.55	7.15	38.30	26.07	38.16	18.98
Variance	1.36	0.68	3.38	5.66	20.94	12.50	14.03	8.17
St. Dev.	1.17	0.83	1.84	2.38	4.58	3.54	3.75	2.86

## Additional Points

*Limitations*. (1) The sample size is relatively small. (2) The studied patellae were of unknown age and gender. (3) The left and right patellae did not belong to the same individual. (4) The study lacks ethnic diversity and data of interethnic comparison. (5) There is a lack of data on the handedness of the studied individuals. Handedness is a major manifestation of cerebral dominance and lateralization patterns. Furthermore, to date, no pertinent research in literature was found to study this aspect. (6) The extent of physical activities of the individuals to whom the patellae belong is unknown. (7) Similarly, the socioeconomic backgrounds of these individuals are unknown, including their nutritional status, growth patterns, and if they had any pathologies or illnesses.
